# Supplementary material for: How to get over with medication errors underestimation? Improving indices of medication errors with focus on intravenous medications in hematopoietic stem cell transplantation setting; a direct observation study
Source: PLoS One. 2024 Aug 22;19(8):e0307753. doi: 10.1371/journal.pone.0307753 (PMC11341051; doi:10.1371/journal.pone.0307753)
Supplement: S1 File — (DOCX) [file pone.0307753.s001.docx]

**Checklist**; (The prepared checklist to document patients’ information, medication administration procedure, and the types of errors)

| Date | Time | Nurse code | Nurse shift | |
| --- | --- | --- | --- | --- |
| Patient | Age | Gender | Height | Wt |
| Drug name | Drug Dose | Dosage form | Administration Rout | |

| 🗹 ***Preparation Error*** |  | Wrong Original Drug Storage  Wrong Diluted Drug Storage  Wrong Reconstituted Drug Storage  Wrong Dilution Volume  Wrong Dilution Solvent  Wrong Reconstitution Volume  Wrong Reconstitution Solvent  Incompatibility |
| --- | --- | --- |
|  |  |  |
|  |  |  |
|  |  |  |
|  |  |  |
|  |  |  |
|  |  |  |
|  |  |  |
| 🗹 ***Administration Error*** |  | Wrong Administration Technique (Rate, Procedure, verification)*  Wrong Time  Wrong Dose  Wrong Dosage Form  Wrong Drug Preparation  Deteriorated Drug  Unauthorized Drug  Extra Dose  Omission of Dose |
|  |  |  |
|  |  |  |
|  |  |  |
|  |  |  |
|  |  |  |
|  |  |  |
|  |  |  |
|  |  |  |

**Description of Error:**

🗹 ***Preparation Error***

- **Wrong Storage**: Drug product incorrectly stored according to manufacturer instruction, which includes:

(1) The drug should be stored out of light or in refrigerator but it is stored otherwise or keeping single dose vials or vials for more than what is manufacturer stated.

(2) The diluted or reconstituted product (for oral solutions or suspensions and injectable drugs) should not be stored at all after preparation or should be stored out of light or in refrigerator but it is stored otherwise

- **Wrong Drug-Preparation Error**: Drug product incorrectly formulated or manipulated (for oral solutions or suspensions and injectable drugs) before administration. This would include;

(1) Wrong dilution volume;

(2) Wrong dilution solvent;

(3) Wrong reconstitution volume;

(4) Wrong reconstitution solvent;

(5) Incompatibility: Physicochemical incompatibility of drugs mixed in the same container or syringe or line and inadequate product packaging.

🗹 ***Administration Error***

- **Wrong Administration Technique Error**: incorrect or omitted action during dose preparation or administration that does not result in another types of error

(1) Administration at the wrong **rate** of administration by intravenous route of a medication to the patient, whatever the technique (direct intravenous, perfusion by gravity or infusion) but patient receives the correct dose,

(2) Administration via **wrong** **procedure** like crushing extended-release forms.

(3) If the prescriber ordered that the patient’s heart rate or blood pressure be determined to fall within certain limits prior to drug administration and this **verification** was **omitted**, this is a wrong technique error (if the HR or BP is measured but and found to be out of the limit but the dose administered, an extra dose error has occurred).

- **Wrong Route:** Medication administered to a patient using a different route than ordered (e.g., oral administration of a drug ordered for I.M. use), or administered via the correct route but at the wrong site (e.g., left eye instead of right).
- **Wrong Time Error**: Administration of medication outside a predefined time interval from its scheduled administration time. It includes administration of a dose more than 60 minutes before or after the scheduled administration time. The 30-minute window was used for medications that were ordered before, with, or after a meal.

Acceptable reasons include situations where the physician has ordered that the patient not consume anything by mouth (NPO), or when the patient is off the floor at a diagnostic test or in surgery.

- **Wrong Dose Error**: Administration to the patient of a dose that is greater than or less than the amount ordered by the prescriber:
  - Any dose that contains the wrong strength or number of preformed dosage units (e.g. tablets);
- For injectable doses, any dose that was ±10% or more of the correct dosage;
- For other dosage forms (e.g. oral liquids), any dose that was ±17% or more of the correct dose in the judgment of the observer;
- For ointments, topical solutions, and similar medications only when the dose was specified quantitatively by the prescriber (e.g., in inches or centimeters of ointment).
- **Deteriorated Drug Error**: Administration of a drug that has expired or for which the physical or chemical dosage-form integrity, has been compromised. This would include, for example, administration of expired drugs and improperly stored drugs.
- **Unauthorized Drug Error** (or **Unordered Drug** or **Wrong Drug**): Administration of a non-prescribed medication to the patient. This would include, for example, a wrong drug, a dose given to the wrong patient, unordered drugs.
- **Extra dose**: An extra dose was any dose given in excess of the total number of times ordered by the physician, such as a dose given on the basis of an expired order, after a drug had been discontinued, or after a drug's administration had been put on hold. If a physician ordered a drug to be given every morning and the nurse gave an additional dose in the evening, the error was placed in this category.
- **Omission Error**: Failure to administer an ordered dose to a patient before the next scheduled dose, it means if no attempt was made to administer the dose, an omission error was counted. Omissions were detected by comparing the medications administered at a given time with doses that should have been given at that time based on the physician's written order.

*Exclusions:* (1) a patient’s refusal to take the medication or (2) a decision not to administer the dose because of recognized contraindications or (3) Doses withheld in accordance with policies for the withholding of medication doses, such as nothing by mouth before surgery. If an explanation for the omission is apparent (e.g., patient was away from nursing unit for tests or medication was not available), that reason should be documented in the appropriate records.
