# Supplementary material for: How to get over with medication errors underestimation? Improving indices of medication errors with focus on intravenous medications in hematopoietic stem cell transplantation setting; a direct observation study
Source: PLoS One. 2024 Aug 22;19(8):e0307753. doi: 10.1371/journal.pone.0307753 (PMC11341051; doi:10.1371/journal.pone.0307753)
Supplement: Attachment — Submitted filename: response to the editor and reviewer.docx [file pone.0307753.s003.docx]

**Journal of PLOS ONE**

Dear Editor,

It is my pleasure to enclose herewith the revised manuscript entitled *“How to get over with medication errors underestimation? Improving indices of medication errors with focus on intravenous medications in hematopoietic stem cell transplantation setting; a direct observation study.”*

We appreciate you and the reviewers for your precious time in reviewing our paper and providing valuable comments. It was your valuable and insightful comments that led to possible improvements in the current version. The authors have carefully considered the comments and tried our best to address every one of them.

We hope the manuscript after careful revisions meet your high standards. The authors welcome further constructive comments if any.

In continue we provide the point-by-point responses. All modifications in the manuscript are showed in track changes.

Sincerely yours,

Dr. Ava Mansouri
Email address: [ava_mansouri_j@yahoo.com](mailto:ava_mansouri_j@yahoo.com)

**Meeting the Journal Requirements:**

1. We have corrected the manuscript based on PLOS ONE's style requirements.

2. It was asked to provide additional details regarding participant consent. We have specified in the ethics statement in the methods, that we obtained the written consent form from patients. We will provide this information as well in online submission information. We also have excluded the minors from our study.

3. We have to state that “The funders had no role in study design, data collection and analysis, decision to publish, or preparation of the manuscript." We have added this statement also to our manuscript

 4. The ethics statement in our manuscript is in under “Compliance with Ethical Standards” in the method section as you have requested

5. We have provided the Excel worksheet of our data in anonymized form and excluded those sheets and parts which were not necessary or included the names’ codes. We will upload it as a supplementary file.

**The response to reviewers’ comments;**

Thank you for giving us the opportunity to submit a revised draft of the manuscript “How to get over with medication errors underestimation? Improving indices of medication errors with focus on intravenous medications in hematopoietic stem cell transplantation setting; a direct observation study.” for publication in the PLOS ONE journal.

We appreciate the time and effort that you and the reviewers dedicated to review our manuscript. We are grateful for the insightful comments on and valuable improvements to our paper. We have considered carefully the comments and addressed most of the suggestions.

We have highlighted the changes within the manuscript with track change and made a point-by-point response to the reviewers’ comments and concerns.

**Reviewer #1**: Dear authors I would like to thank you for the nice and very important topic you had explored but I have the following comments for further improvement:

**Authors answer**; thank you, we are so grateful for your interest in our manuscript and your valuable comments

**1- The abstract should not contain abbreviations so please remove all and advice to read journal requirements for submission.**

Authors answer; the Changes are applied as reviewer suggested

**2- The abstract should be divided under subtitles (introduction, methods, results and discussion) so pleas restructure your abstract.**

**Authors answer**; the Changes are applied as reviewer suggested

**3- The references at the end of cited sentences should be inside brackets [ ] not ( ), so please modify all.**

**Authors answer**; the Changes are applied Changes are applied as reviewer suggested

4- **I have one major concern and really you need to explain that very well in your manuscript, how the results were validated? I mean did you validate the observations that were conducted by one person which are more prone to bias, if yes how ? if not why you didn't that and how you will overcome this major issue?**

**Authors answer**; Thank you for pointing out this important issue. We are totally aware of the significance of the concern you have raised. Actually we had conducted a first round study, following all the steps and under the same conditions as the main study. Unfortunately we have thought that this might not be essential due to amount of the details. Therefore, here we will explain the whole process and will also include it in the method due to yours and other reviewers’ reasonable concern;

Initially, the observer underwent training by the clinical pharmacist professor, who oversees all three BMT wards. Subsequently, the observer utilized the prepared checklist and conducted a pilot study in the relevant wards. The observer diligently documented information from patients' charts, recorded her observations, and completed the checklists. Following this, she presented the gathered information to the study supervisor, a Pharm.D pharmacist, who independently filled out additional checklists based on the observer's findings.

Subsequently, a comprehensive comparison of the completed checklists was undertaken by the team, comprising the clinical pharmacist professor in charge, the responsible oncologist of the wards, the study supervisor, and the observer. Any discrepancies in the errors detected by the observer and the supervisor were categorized as true, false positive, and false negative. They also deliberated on the classification of the errors. Subsequently, necessary adjustments were made to the observation process and error detection. Additionally, during the primary study phase, some observations were randomly verified by the supervisor.

**Reviewer #2**: There are a few points that are necessary to be considered

1. **This study is cross-sectional observational study, while the study was carried out between 2014-2015;**

**Authors answer**; The study was cross-sectional lasting from 2014 to 2015, and there was no follow up afterward.

2. **The data were collected from three wards, related to Teaching hospital. The possibility of bias is high**

**Authors answer**: We agree with the reviewer’s assessment. Thank you for your concern and pointing out this important issue, here we have your answer as we explained in response to other reviewers;

Actually, we conducted a preliminary study, following all the steps and under the same conditions as the main study. Regarding your concern, we explained the whole process here and in the methods section.

Initially, the observer underwent training by the clinical pharmacist professor, who oversees all three BMT wards. Subsequently, the observer utilized the prepared checklist and conducted a pilot study in the relevant wards. The observer diligently documented information from patients' charts, recorded her observations, and completed the checklists. Following this, she presented the gathered information to the study supervisor, a Pharm.D pharmacist, who independently filled out additional checklists based on the observer's findings.

Subsequently, a comprehensive comparison of the completed checklists was undertaken by the team, comprising the clinical pharmacist professor in charge, the responsible oncologist of the wards, the study supervisor, and the observer. Any discrepancies in the errors detected by the observer and the supervisor were categorized as true, false positive, and false negative. They also deliberated on the classification of the errors. Subsequently, necessary adjustments were made to the observation process and error detection. Additionally, during the primary study phase, some observations were randomly verified by the supervisor.

**3. The results were presented as descriptive analysis, while the inferential analysis between the three wards were missed**

**Authors answer**; thank you for pointing this issue out.

We have conducted a comparison between wards. Although we will include the results in the relevant section, we will also provide the explanation here. The characteristics of the wards were the same; they all had 9 beds with a nurse-patient ratio of 1 to 4 or 1 to 5. We also analyzed the characteristics of the nurses and could not find any significant differences between their characteristics and the medication errors they were involved in. Although we found that error rates varied significantly between wards, the close range of their CTOEs (24 to 31) made it clinically unimportant.

4. I**t is difficult to get a conclusion from data obtained from specific wards**

**Authors answer**; thank you for sharing your concern. However, as we mentioned before, our primary objective was to identify the most effective approach for reporting medication errors that occur in the BMT wards of a hospital, specifically for patients undergoing HSCT. We aimed to achieve this through a well-defined, precise detection method, using a denominator and checklist. This reporting method offers superior clarity and provides more detailed information through its indicators. Consequently, it serves as a valuable guide for developing targeted preventive measures and assessing their effectiveness. It is crucial to investigate the applicability of this method in other settings to fully understand its significance and relevance.

**5. Check the references: typing errors**

**Authors answer**; Changes are applied as reviewer suggested

**6. This study that was done in 2014-2015 will not add new information in 2023**

**Author response:** While we appreciate the reviewer’s feedback, we respectfully disagree. We think this study makes a valuable contribution to the field because the issue that we raised and took action to solve is still an important issue as well. Because the reporting of medication error articles is still done in an old-fashioned way and total opportunities for error (TOE) is still common and established. Our goal is to review TOE, which is still routine in 2023.

**Reviewer #3:** The manuscript technically sound, and the data support the conclusions. The statistical analysis has been performed appropriately. The conclusions drawn appropriately based on the data presented. The manuscript presented in clear and comprehensible fashion

**Author response:** thank you, we are so grateful for your time and effort. We are also very pleased that you find the manuscript interesting as the way it is.

**Reviewer #4:** After reviewing the current manuscript:

1- The paper is well written.

2- All references are updated.

3- The authors discussed the main idea successfully.

I recommend the acceptance of the paper in its current form.

**Author response:** We are so grateful for your valuable time and assessment and we are so pleased that you already find the manuscript interesting as it is.

**Reviewer #5**: Major Points and Feedback:

- Data Collection Tools and Analysis:

**The use of SPSS-21 for data analysis is well-suited for this type of study. The choice of using Excel for data input is reasonable; however, more details on how data were cleaned and processed before analysis would be useful.**

**Author response:** Thank you for this suggestion. The observer recorded the data and error details from the checklists into Excel after each observation. The columns included the following information: ward name, nurse code, observation number, date, morning/evening shifts, drug generic name, drug brand name, dosage forms (powder or solution), dosage unit, number of administrations in one day (QD, BD, TDS, QOD). The subsequent columns were dedicated to error types, with the following coding: 1 - correct checkpoint, 2 - incorrect checkpoint (error). In cases where errors occurred, such as monitoring or exposure to light during administration, there were three possible conditions: 1 - not relevant for that drug, 2 - should be considered and done correctly, 3 - should be considered and not done (error). Finally, the demographic data of the nurses for each observed drug were entered into the last columns. I hope this explanation is satisfactory and provides sufficient clarity.

•	Descriptive Statistics:

The presentation of data through descriptive statistics such as mean, standard deviation, frequency, and percentage is well executed. This aids in understanding the spread and central tendencies of the data.

Total Opportunities for Errors (TOE) & Corrected Total Opportunities for Errors (CTOE):

**The distinction between TOE and CTOE is a crucial one and offers a novel approach. The authors should consider focusing more on explaining the real-world implications of this distinction. How does CTOE improve upon existing error rate measurements?**

**Author response:** thank you for pointing this topic out. CTOE can be accounted as standardized error rate. Since the drugs have different characteristics and their share in total error occurrence is absolutely different. CTOE can reveal the real causes and largest share of errors in the drug utilization process (both in the terms of drugs and neglected stages)

•	Results Section:

**The results are detailed and cover a broad range of observed data. However, it may be helpful to provide more context or a brief summary before diving into specific numbers.**

**Author response:** the Changes are applied as reviewer suggested

**It would be beneficial to discuss why certain drugs, like Imipenem+cilastatin, had high error rates. Are these errors due to the complexity of the drug's administration, or are there other factors at play?**

**Author response:** Thank you for pointing this out. While we acknowledge the importance of this consideration, it is beyond the scope of this manuscript as our study design and method are not suitable for determining the causes of medication errors. We are only able to present which medications have what types of errors and their prevalence. Additionally, imipenem+cilastain had a high number of errors, but it was not the most erroneous drug overall based on CTOE. As we previously reported, cyclosporine was the most erroneous medication in total based on CTOE, meaning it had the highest number of errors in the BMT wards, which we have discussed in the erroneous drugs section of our discussion.

Tables:

**Tables are well-presented, offering a breakdown of errors, observations, and characteristics. However, using clearer labels and perhaps footnotes to explain terms such as SPE, CTOE, etc., directly in the table can enhance readability.**

**Author response**; We have tried to make it more clear and I hope your request is resolved.

Potential Limitations:

**The study observes only 25 nurses across three wards. A discussion on the representativeness of the sample and any potential biases in observation would be beneficial.**

**Authors answer:** We totally agree with the reviewer’s assessment. Thank you for your concern and pointing out this important issue, here we have your answer as we explained in response to other reviewers earlier;

Actually we had conducted a first round study, following all the steps and under the same conditions as the main study. Unfortunately we have thought that this might not be essential due to amount of the details. Therefore, here we will explain the whole process and will summarize it in the method due to yours and other reviewers’ reasonable concern;

At first, the observer was trained by the clinical pharmacist professor who is in charge of all the tree BMT wards. Then the observer referred to the related wards with the prepared checklist and did a test study in the field. The observer has recorded all the information from patients’ chart as well as all of her observations and filled out the checklists. Afterward she delivered the gathered information to the study supervisor (a Pharm.D pharmacist) and the supervisor also filled the checklists dependently based on the information that observer provided. Afterward a thorough comparison between the completed checklists was done by the team of the in charge clinical pharmacist professor, the responsible oncologist of the wards, the study supervisor and the observer. The diversity between the observer and the supervisor regarding the detected errors were categorized as; true, false positive and false negative. In addition they have discussed where the errors belong (in which categories). The necessary modifications made to the observation process and errors detection. During the main study phase some of observations were randomly checked by the supervisor as well.

We have also done the comparison between wards, we now also add it in the results part. The ward characteristics were the same; they all have 9 beds with nurse patient ratio 1 to 4 or 1 to 5. We also analysis the characteristics of nurses and we could not find any significant differences between their characteristics and medication errors they have engaged. Although we have found that error rates were significantly different between wards but since their CTOEs are close and ranged between from 24 to 31, it was not clinically important.

**The paper could delve deeper into the implications of the fact that all observed nurses were female and held a Bachelor of Nursing degree.**

**Author response:** Thank you for mentioning this point. Although we agree that this can be an important issue, but it is beyond the scope of this manuscript , since talking about these characteristics that you are pointing out, is lying in the concept of causes of errors. As far as it was within the scope of our study, there were no significant difference between the nurses’ characteristics (included their degree) and errors that they engaged.

Recommendations for Improvement:

**The introduction of the concept of CTOE should be supported by further literature. If this is a novel concept introduced by the authors, a more in-depth rationale behind its introduction would be beneficial.**

**Author answer;** Thank you for bringing this issue up. We have mentioned in the manuscript that; “We developed and defined CTOE for a more accurate and specific evaluation of the scope of medication errors”, so in response to your question this is a novel concept. We have also tried to explain the concept in the method. In addition we explain and support the CTOE concept in our discussion using our own result again, which make it more clarifying and is more rationalized.

**A discussion or conclusion section summarizing key findings and their implications would add value.**

**Author answer;** thanks to your great suggestion, we have now added some more summarized results in 1^st^ part of our discussion.

**Recommendations for reducing error rates based on the findings would also be insightful.**

**Author answer;** we appreciate your valuable suggestions. We have tried our best to recommend based on our finding in the scope of our study which is using of CTOE in other different settings and different types and medications routes to evaluate if it is practical, also we have recommended further studies in BMT to use CTOE along with implementing preventive measures and assess their effectiveness, feasibility, and costs.

**It might be useful to compare the findings with similar studies (if any) to provide a benchmark or context to the observed error rates.**

**Author answer;** Thank you for your suggestion. As you have asked we totally explain what studies or results have we made comparison whit and why in the discussion. We made some changes based on your comment, in case it was not clear, and I will refer to it here in case it might be hard to find;

“Since there are no other direct observation studies in this setting, it is challenging to compare our results. However, a systematic review published in 2019 on MEs in BMT evaluated 11 studies, including eight case reports and three studies on narrow topics related to BMT [30]. The review generally reported that most errors occurred during the administration and prescription stages.

Although chemotherapy wards are similar to BMT wards in many ways, it was even more challenging for us to identify errors in the chemotherapy inpatient setting. This difficulty arose due to the limited number of studies available on this topic, as well as the fact that direct observation was not employed as a method in any of these studies. Furthermore, none of the studies specifically focused on evaluating medication errors (MEs) in the intravenous (IV) process or reported errors related to the IV process separately. Therefore, in the discussion, we would discuss our results in conjunction with similar studies for each aspect. However, in the absence of similar studies, we will attempt to explain and justify our results by referencing other relevant studies in the field. By adopting this approach, we aim to provide a comprehensive understanding of the subject matter despite the limitations in the available literature.

We also have tried to explain before each part that how we would compare our results in that part.

Conclusion:

The paper presents a comprehensive analysis of medication errors in drug preparation and administration, introducing novel concepts to enhance understanding. While the methodology and presentation of results are rigorous, there are areas for improvement in explanation, context, and discussion. With further refinement, this paper can serve as a significant contribution to the field of medication error research.

**Reviewer #6: I only have one major comment about the study. The authors mentioned that a final year pharmacy student served as the observer, who compared their notes with the medical chart, order sheet, and nurses' notes every day. Is it possible that the observer could make errors and have the authors conducted any reliability analysis?**

We totally agree with the reviewer’s assessment. Thank you for your concern and pointing out this important issue, here we have your answer as we explained in response to other reviewers;

Actually we had conducted a first round study, following all the steps and under the same conditions as the main study. Unfortunately we have thought that this might not be essential due to amount of the details. Therefore, here we will explain the whole process and will summarize it in the method due to yours and other reviewers’ reasonable concern;

At first, the observer was trained by the clinical pharmacist professor who is in charge of all the tree BMT wards. Then the observer referred to the related wards with the prepared checklist and did a test study in the field. The observer has recorded all the information from patients’ chart as well as all of her observations and filled out the checklists. Afterward she delivered the gathered information to the study supervisor (a Pharm.D pharmacist) and the supervisor also filled the checklists dependently based on the information that observer provided. Afterward a thorough comparison between the completed checklists was done by the team of the in charge clinical pharmacist professor, the responsible oncologist of the wards, the study supervisor and the observer. The diversity between the observer and the supervisor regarding the detected errors were categorized as; true, false positive and false negative. In addition they have discussed where the errors belong (in which categories). The necessary modifications made to the observation process and errors detection. During the main study phase some of observations were randomly checked by the supervisor as well.

We have also done the comparison between wards, we now also add it in the results part. The ward characteristics were the same; they all have 9 beds with nurse patient ratio 1 to 4 or 1 to 5. We also analysis the characteristics of nurses and we could not find any significant differences between their characteristics and medication errors they have engaged. Although we have found that error rates were significantly different between wards but since their CTOEs are close and ranged between from 24 to 31, it was not clinically important.
