# Supplementary material for: How to get over with medication errors underestimation? Improving indices of medication errors with focus on intravenous medications in hematopoietic stem cell transplantation setting; a direct observation study
Source: PLoS One. 2024 Aug 22;19(8):e0307753. doi: 10.1371/journal.pone.0307753 (PMC11341051; doi:10.1371/journal.pone.0307753)
Supplement: Attachment — Submitted filename: response to the reviewers.docx [file pone.0307753.s004.docx]

The response to reviewers’ comments;

Thank you for giving us the opportunity to submit a revised draft of the manuscript “How to get over with medication errors underestimation? Improving indices of medication errors with focus on intravenous medications in hematopoietic stem cell transplantation setting; a direct observation study.” for publication in the PLOS ONE journal.

We appreciate the time and effort that you and the reviewers dedicated to review our revised manuscript. We are grateful for the insightful comments on and valuable improvements to our paper. We have considered carefully the comments and addressed most of the suggestions.

We have highlighted the changes within the manuscript with track change and made a point-by-point response to the reviewers’ comments and concerns. Those track changes that are not highlighted are the edited parts by the native English speaker.

**Reviewer #8: Dear authors I appreciate the efforts in revising the manuscript as recommended by the reviewers. I strongly recommend the manuscript is given to a native English user/speaker to correct all grammatical errors and break down winding sentences for conciseness and easier comprehension.**

**Authors answer**; we are grateful for the time and effort you spent to review our manuscript. We are so pleased that you are satisfied with the manuscript revision and we have followed your suggestion about the native English editing

**Reviewer #9: Thank you for this clinical relevant study. I have organised my feedback into major and minor comments as below**

Major

**a. Generic**

**1- Please avoid subjective language, such as "most defined," "great help," and "poor." English editing before resubmitting is recommended.**

**Authors answer;** Thank you for pointing out this important issue. The manuscript is now edited by a native English speaker.

**b. Abstract**

**1-The definition of CTOE is provided, but the terms TOE and Proportional Error Ratio need clarification as well.**

**Authors answer;** Thanks to your comment, the definitions are now clarified

**2- The presentation of figures such as 2.98 or 298% is unclear without context. Additionally, sometimes the inconsistent mention of CTOE and SPE and the absence of data for cyclosporine are confusing. I would recommend only using the most important indicators in the abstract.**

**Authors answer;** We have considered your recommendation and the changes are applied as reviewer suggested

**c. Introduction**

**1-The mention of HSCT seems abrupt. Please restructure the paragraph to highlight the differences between HSCT settings and general settings.**

**Authors answer;** We have revised the text to address your concerns and hope it is now clearer.

**2-It is unclear what is being compared when mentioning the benefits of preventive ME interventions. Please specify.**

**Authors answer;** we are apologizing for the confusion, we have tried to say if we want to demonstrate the benefits of preventive ME interventions, we should be able to compare these interventions. We also have corrected this misleading statement in the manuscript.

**3-The potential barriers in epidemiology studies within this field require clarification to strengthen the introduction.**

**Authors answer;** thank you for pointing out this issue**,** we have tried to make it understandable

**4-The calculation of 16-365 times is unclear, as the reference does not directly mention these figures.**

**Authors answer;** you are absolutely right, these numbers are not directly reported in the reference *“Flynn EA, Barker KN, Pepper GA, Bates DW, Mikeal RL. Comparison of methods for detecting medication errors in 36 hospitals and skilled-nursing facilities. Am J Health Syst Pharm. 2002;59(5): 436-46.”*, we calculated them ourselves as we compare number of errors detected by each method. We extract these from the text of the cited article; “Direct observation detected 300 of these errors and 73 false positives, which produced an error rate of 14.6%. For the same doses, chart review detected 17 of the 457 errors and 7 false positives, yielding an error rate of 0.9% (14.6/0.9=16), while incident report review detected only 1 error for an error rate of 0.04% (14.6/0.04=365).”

**Please also clarify the difference between these methods.**

**Authors answer;** we tried to explain methods in a concise form as you have asked.

**5-The significance of denominators is mentioned, but not in the context of the study's objectives. I am not sure if I can understand what are you suggesting,**

**Authors answer;** we appreciate your concern and hopefully we may well make it more understandable with the following explanation; We have tried to find and establish a denominator in reporting ME rates (since the nominators are detected errors in whole or part of it in all MEs studies thus the main problem here is denominators), so that we can overcome the underestimation of MEs, misunderstanding of MEs and the share of responsible medications. That is why we are highlighting denominators role.

**6- A more concise introduction is recommended for clarity.**

**Authors answer;** we thank you for pointing out this problem, the study is a little complicated that’s why we try to address every necessary things in our manuscript to make it rationality understandable, but we have revised it as much as possible to make it more concise.

**d. Method**

**1 Briefly describe the "disguised direct observation method" based on Barker and McConnell's practice, including its performance in prior research.**

**Authors answer;** the Changes are applied as reviewer suggested and they are now added to the text

**2-The rationale for selecting the 32 medications observed should be clarified.**

**Authors answer;** as we have mentioned in the method, these 32 IV medications are the most used medications in the HSCT wards reported by the nurses’ supervisor, so we can exclude other not so usual medications which nurses might not be very familiar with their IV administration process. Thanks to your comments, it is now added to the manuscript.

**3- Details on how random verification was performed are needed.**

**Authors answer;** the Changes are applied as reviewer suggested and they are now added to the text

**4- The reasoning behind the relative error checkpoints for each drug could be elaborated in the Supplement.**

**Authors answer;** as you have requested, all the details are assigned as supplement to the manuscript and sent to the journal previously

**e. Results**

**1-Table 1's format is unclear, particularly regarding the overall characteristics and the association between Ward A and nursing errors.**

**Authors answer;** We are sorry for the confusion, this table is only representing the number of each characteristic and there is no association in this table. We have made it clearer in the text and table.

**2 The definition regarding error rates per SPE belongs in the Methods section. Please define these indicators clearly and consistently in the Method and straight use the term, for example, CTOE in the Result section.**

**Authors answer;** thanks to your consideration, we have defined each error rate in method completely individually and we have revised the result as you were suggested

**3 The abrupt presentation of nurse characteristics and error differences between wards needs to be reorganised.**

**Authors answer;** we have applied the changes in reports order as reviewer suggested **f. Discussion**

**1-Restructure the Discussion to outline main findings, compare with prior research, and discuss strengths and limitations. A significant rewrite is needed for clarity.**

**Authors answer;** the changes are applied as reviewer suggested and the discussion has been rewritten

**2-Please highlight the value of the CTOE at the beginning of the discussion.**

**Authors answer;** thanks for your consideration the CTOE value is now added at the beginning of the discussion

**3-The argument against direct comparison lacks new insights and needs strengthening.**

**Authors answer;** thank you for sharing your concern. As we mentioned before, the issue that you are pointing out might be due to limited number of studies available on this topic, as well as the fact that direct observation was not employed as a method in any of these studies, besides the fact that none of the studies specifically focused on evaluating MEs in the IV process or reported errors related to the IV process separately. But we hope this issue is somehow resolved after the discussion is rewritten and edited.

**Minor Comments

a. Introduction
1-Please keep consistency when discussing dose errors, specifying if it relates to IV medication or dose errors.**

**Authors answer;** the Changes are applied as reviewer suggested

**2-Once the abbreviation is defined, please use abbreviations consistently.**

**Authors answer;** the corrections are as reviewer suggested

**3 Full terms should be used for clarity, e.g., "denominators of xxx" instead of "denominators."**

**Authors answer;** this is corrected as reviewer suggested

**b. Method
1-Please use "sex" instead of "gender" for biological distinctions.**

**Authors answer;** the Changes are applied as reviewer suggested

**2-Please specify which author conducted the observations in each steps.**

**Authors answer;** the correction is applied as reviewer suggested **c. Results**

**1-Please clarify the calculation of reported numbers, including numerators and denominators.**

**Authors answer;** all of the denominators and numerators are defined in the method completely
 **d. Discussion
1 Please improve readability by correctly formatting references, as the current method (e.g., "33 et al.") is incorrect.**

**Authors answer;** we are apologizing for this mistake, it is corrected now and the manuscript is given for native English editing

We would like to thank the referee again for taking the time to review our manuscript.

**Reviewer #10: Thank you for the opportunity to review the article. I'm pleased to inform you that I find the article suitable for publication. The research conducted is rigorous, the findings are significant, and the writing is clear and engaging.**

**Authors answer;** thank you for your time and effort to review our manuscript. We are so pleased that you are satisfied with our revision.
